# Differential Impact of Specific Amino Acid Residues on the Characteristics of Avian Influenza Viruses in Mammalian Systems

**DOI:** 10.3390/pathogens11111385

**Published:** 2022-11-19

**Authors:** Dayly Mashaal, Sara H. Mahmoud, Christin Müller, Noura M. Abo Shama, Amal Abo Kamer, Ahmed A. Abdelaziz, Mohamed A. Ali, Stephan Pleschka, Ahmed Mostafa

**Affiliations:** 1 Pharmaceutical Microbiology Department, Faculty of Pharmacy, Tanta University, Tanta 31527, Egypt; 2 Center of Scientific Excellence for Influenza Viruses, National Research Centre, Giza 12622, Egypt; 3 Institute of Medical Virology, Justus Liebig University Giessen, Schubertstrasse 81, 35392 Giessen, Germany; 4 German Center for Infection Research (DZIF), Partner Site Giessen-Marburg-Langen, 35392 Giessen, Germany

**Keywords:** mammalian-adaptive marker, 591K, 627K, H5N1, H9N2

## Abstract

Avian influenza virus (AIV) H9N2 was declared to be endemic in birds of the Middle East, in particular in Egypt, with multiple cases of human infections. Despite concerns about the pandemic threat posed by H9N2 AIV, due to the fact that its receptor specificity is similar to that of human influenza viruses, its morbidity and mortality rates in humans are so far negligible. However, the acquisition of specific adaptive amino acid (aa) mutations in the viral polymerase can enhance cross-species transmission of the virus itself or of reassortants, which gained these changes. The polymerase basic protein 2 (PB2) is one of the key determinants for AIV adaptation towards mammals. Although mammalian pathogenicity-related mutations (MPMs) in PB2 genes were identified in different AIVs, the specific effect of single or multiple mutations on viral fitness has not been compared so far. Here, we studied the effect of the aa K at position 591, which was frequently reported in the PB2 of Egyptian H9N2 isolates, on the proliferation efficiency and polymerase activity of an H5N1 (clade 2.2.1.2) AIV already carrying the mammalian adaptive mutation 627K. Using reverse genetics, we generated a set of recombinant parental strains and H5N1 variants carrying the avian-like 591Q/627E or mammalian-like adaptive mutations 591K/627K (H5N1_EGY_, H9N2_EGY_, H5N1_PB2-H9N2EGY_, H5N1_H9N2_PB2_K591Q_, H5N1_PB2_K627E_, H5N1_PB2_K627E/591K_, H5N1_PB2_627K/591K_). Regardless of the avian-like 627E or the mammalian-adaptive 627K, both variants carrying the 591K (H5N1_PB2_K627E/591K_, H5N1_PB2_627K/591K_) and the reassortant H5N1_PB2-H9N2EGY_ replicated to significantly higher levels in mammalian continuous MDCK and Calu-3 cell lines and primary normal human bronchial epithelial cells than the parental H5N1_EGY_ virus (carrying solely the 627K adaptive mutation). Expectedly, the H5N1 variants carrying avian-like PB2 mutations (H5N1_H9N2_PB2_K591Q_, H5N1_PB2_K627E_) replicated to significantly lower levels than the parental H5N1_EGY_ virus in the predefined primary and continuous mammalian cell line systems. Consistently, the activity of H5N1 subtype AIV polymerase complexes comprising PB2 segments with singular 591K or combined with 627K was significantly enhanced when compared to parental H5N1_EGY_ and H9N2_EGY_. This study emphasizes the significant impact of 591K containing PB2 segments in the background of H5N1 polymerase on viral fitness in addition to the well-known MPM 627K in vitro.

## 1. Introduction

The three polymerase subunits of influenza A viruses (IAV), PB1, PB2, and PA, comprise the heterotrimeric viral RNA–dependent RNA–polymerase (RdRp), which is responsible for transcription and replication of the viral RNA (vRNA) genome [1]. Briefly, the PB1 subunit is the linker between PB2 and PA through its N-terminal interaction with the C-terminus of PA and the C-terminal interaction with the N-terminus of PB2 [2]. Whereas PB1 is well-known to play a key role in polymerase activity, the PB2 subunit has a 5′ cap-binding activity targeting host mRNAs to initiate IAV genome transcription. Furthermore, structural and functional studies revealed the PA key role in the endonucleolytic cleavage of host mRNAs, suggesting a role as a multifunctional protein [1,3,4].

As a complex genomic entity with a unique structure and function, the IAV ribonucleoprotein complex (vRNP) is composed of the RdRp subunits as a globular head linked to rod-shaped structures of the vRNAs folded on the viral nucleoprotein (NP) [1,5].

Unlike most other RNA viruses, the replication of the influenza viral genome depends on the nuclear functions of the host cell. RNPs containing the negative sense vRNAs of the eight viral segments are imported into the nucleus of the infected cell. The vRNAs act as templates for the synthesis of the corresponding capped and polyadenylated mRNA transcripts. The replication of the vRNAs is initiated by generating complementary positive sense RNA copies (cRNAs), which are subsequently used as a template to synthesize more vRNAs.

To prime the viral transcription of the vRNA, the viral RdRp utilizes the cap structure of premature cellular mRNAs. During this process, the PB2 subunit of the RdRp recognizes and binds to the 5′ cap structure (m7GpppX) of nascent cellular mRNAs and the PA cleaves the PB2-bound cap structure approximately at 10–13 nucleotides from their 5′-ends by virtue of its endonuclease activity [1]. This process, which is carried out by PB2 and PA to pinch off the 5′ cap structure of cellular mRNAs, is known as “cap-snatching”.

A breakthrough that allowed for detailed comprehension of the vRNP structure, to deduce the mechanisms of RNA replication and transcription, the intracellular trafficking of the viral genome, selective packaging of the vRNPs, and viral gene reassortment, was the development of in vitro reverse genetic (RG) systems and minigenome assays for IAV [5,6]. The RG approach to IAV is based on the eight different genomic vRNAs cloned as cDNAs into specific plasmids, from which new vRNA-like transcripts can be expressed to form biologically active RNPs together with transiently expressed RdRp subunits and NP, allowing the rescue of an entire and genetically accessible infectious virus.

Substitutions of polymerase subunits have been reported to alter the replication, pathogenicity, and host range of IAV in mammals and poultry [7,8,9,10]. Concerning the PB2 protein of avian influenza viruses (AIV), a glutamic acid replacement at position 627 with lysine (E627K) is known as an important host range determinant, which enhances efficient viral genome replication at mammalian-like temperature, transmissibility, and pathogenicity of AIV to mammals [11,12,13]. For instance, human IAV prefers a temperature of approximately 33 °C to replicate in the upper respiratory tract (URT), whereas the AIV prefers 41 °C to replicate in the intestinal tract. Contrary to AIVs with 627E in the PB2, AIVs carrying an E627K mutation can replicate efficiently at 33 °C in the URT of mammals [14]. The absence of the human signature (K) at position 627 is often compensated with a D701N mutation to retrieve higher replication, transmissibility, and virulence of AIVs in mammals [15]. Both the 627K and 701N human signature markers have been found in most human isolates of H7N9 causing human infections and fatalities in China in 2013 [16]. In contrast, the pandemic 2009 H1N1 virus is missing the human signature at position 627 (627K) or 701 (701N), but it is efficiently replicating and transmitting in mammals due to two compensatory mutations, 590S and 591R, which maintain the human signature [17,18].

The co-circulation of zoonotic highly pathogenic avian influenza virus (HPAIV) of the H5N1 subtype and AIV of the H9N2 subtype in Egypt makes this country a hypothetical hot spot for the emergence of reassortant, phenotypically altered viruses [19]. Moreover, we have recently shown that the PB2 segment of an Egyptian H9N2 AIV (PB2_H9N2EGY_) encoding the mammalian 591K signature could enhance the replication efficiency of an H5N1 type AIV more than the parental PB2 segment carrying the 627K mammalian adaptive marker. Herein, we investigated the differential impact of 627K and 591K mutations on the characteristics of AIVs in mammalian systems.

## 2. Materials and Methods

### 2.1. Cells, Viruses, and Plasmids

The Madin–Darby canine kidney (MDCK) cells, human embryonic kidney cells (293T), and cultured human airway epithelial cells (Calu-3) were maintained in Dulbecco’s modified Eagle medium (DMEM) (Invitrogen, Germany) containing 100 I.U./mL penicillin/100 μg/mL streptomycin (P/S) and 10% fetal bovine serum (FBS). Cryopreserved normal human bronchial epithelial (NHBE) cells were obtained from Lonza and were seeded on trans well plates (Corning Costar, CLS3470-48EA) coated with collagen IV (Invitrogen) and grown in a mixture of DMEM (Invitrogen) and BEGM (Lonza, CC-3170) supplemented with retinoic acid (75 nM). Fresh medium was added regularly after 2 days [9,20]. After reaching confluence, the cells were cultivated for differentiation under air–liquid conditions for four additional weeks. The medium from the basolateral compartment was renewed every 2–3 days, and the apical surface was washed every week with PBS (Invitrogen). All cell monolayers were incubated at 37 °C in a humidified 5%CO_2_ incubator.

For reverse genetics, complete sets of pMP*ccd*B plasmids encoding the eight viral segments virus A/chicken/Egypt/N12640A/2016(H5N1_EGY_) and the low pathogenic virus A/chicken/Egypt/S12568C/2016(H9N2_EGY_) were generated as previously described [9]. The mutated PB2 segments were constructed using the previously generated PB2 plasmids of H5N1_EGY_ and H9N2_EGY_ as templates by site-directed mutagenesis (Agilent, USA) as previously described [9] using specific mutagenesis primers (Table 1).

### 2.2. Generation of Reassortant-, Mutant-, and Wild-Type Strains

To generate wild-type H5N1_EGY_ and H9N2_EGY_ strains, H5N1_EGY_ reassortant expressing PB2 protein of H9N2_EGY_, and other H5N1_EGY_ mutants that carry the predefined avian-like and mammalian-like mutations (Table 2), 8 μg of plasmid DNA (1 μg of each plasmid) encoding different combinations of the eight viral segments were transfected into a co-culture of 293T/MDCK-II cells in a ratio of 3:1 as previously described [9]. Approximately 8 h post-transfection, the transfection mixture was replaced with fresh Opti-MEM-reduced serum medium, supplemented with 0.2% bovine serum albumin (BSA), 1% pen/strep, and 1 µg/mL of L-(tosylamido-2-phenyl) ethyl chloromethyl ketone (TPCK)-treated trypsin. About 72 h post-transfection, the supernatant of the transfected cells was harvested, and an aliquot of 100 µL of each supernatant was used to inoculate specific-pathogen-free (SPF) embryonated eggs for 36 h. The rescued viruses were harvested and titrated using the plaque infectivity assay and stored in small aliquots at −80 °C until use.

### 2.3. In Vitro Replication Efficiency of Reassortant and Wild-Type Strains

MDCK and Calu-3 cells were cultured in 6-well plates until 80–90% confluent cell monolayers were formed and then infected in triplicate in phosphate-buffered saline (PBS) containing 0.2% bovine serum albumin (BSA), 1 mM MgCl_2_, and 0.9 mM CaCl_2_ at a multiplicity of infection (MOI) of 0.1 in a humidified 5%CO_2_ incubator at 37 °C with each wild-type, reassortant, and mutant strain for 1 h to allow viral adsorption. Following viral adsorption, the inocula were removed and replaced with infection media (DMEM containing 0.2% BSA, 1%P/S, and 1 µg/mL TPCK-treated trypsin). The plates were incubated at a humidified 5% CO_2_ incubator at 37 °C for 36 h. An aliquot of 100 µL was harvested at 6, 12, 24, and 36 h post-infection (h p.i.). At the same time, differentiated NHBE cells were infected with the predefined viruses at a MOI = 1 to allow efficient infection of the primary cells. Supernatants were collected at 6, 12, 24, and 36 h p.i. and titrated using a focus assay as previously described [21].

### 2.4. Luciferase Reporter Assay

The luciferase reporter gene assay was performed as previously described [9,10,22] with minor modifications. Briefly, 293T were cultured into 6-well plates and co-transfected with a mixture of the following: (1) 1 µg of each pMP*ccd*B expression plasmid encoding individual vRNP complex components for the parent, reassortant, and variant strains; (2) the vRNP-expressing plasmids were mixed with 40 ng Renilla luciferase expression plasmid (pRL-SV40) for normalization; and (3) 200 ng of a firefly luciferase reporter plasmid pHW72-Luc expressing negative-sense firefly luciferase flanked by noncoding sequences (NCR) of NS segment under polymerase I promoter control. The plasmid DNA mixture was then transfected into 293T cells using Trans-IT2020 (2 µL/µg) for 8 h as previously described [21,23]. Subsequently, the transfection mixture was replaced with 1x Opti-MEM with 0.2% BSA and 1% pen/strep and kept in a humidified 5% CO_2_ incubator at 37% for 24 h. The transfected cells were then harvested, washed once with 1x PBS, and then lysed using 200 L of “1x passive lysis buffer” (Promega, Madison, Wisconsin, USA). The Dual-Luciferase Reporter Assay System (Promega) and a Spark 10M multimode microplate reader were then used to quantify the Firefly/Renilla luciferases as Relative Luminometer Units (RLU, standardized to Renilla luciferase). RLU corresponds to the fold induction of polymerase activity.

### 2.5. Ethical Statement and Biosafety

All experiments using infectious viruses were performed in accordance with the Egyptian and German biosafety regulations pertaining to the propagation of influenza viruses. All experiments involving IAVs were performed using Biosafety Level 3 containments approved for such use by local authorities (NRC, Dokki, Egypt).

## 3. Results

### 3.1. Genetic Compatibility among Wild-Type and Mutated PB2 Segments in the Genetic Background of H5N1_EGY_

**Figure 1:**
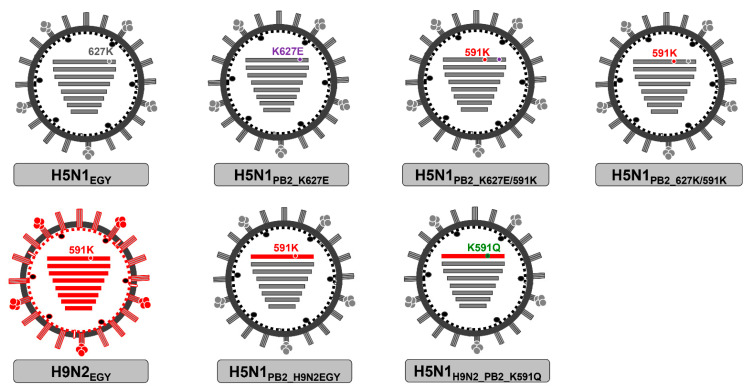
Viruses generated to study the differential impact of 591K and 627K in PB2 segments of H5N1 (grey) and H9N2 (red) viruses. The wt and mutated segments of all viruses were compatible and could be successfully rescued.

Following the insertion of PB2 mutations of interest, wild-type H5N1_EGY_ and H9N2_EGY_, reassortant H5N1_PB2_H9N2EGY_ and H5N1_PB2_H9N2EGY_K591Q_, and mutated H5N1_PB2_K627E_, H5N1_PB2_K627E/591K_, and H5N1_PB2_627K/591K_ were successfully generated and propagated in SPF embryonated eggs (Figure 1). Interestingly, every reassortant could be rescued, demonstrating that such viruses could principally emerge in nature.

### 3.2. The Adaptive 591K Can Compensate for the 627K in the Genetic Background of H5N1_EGY_ in Different Mammalian Systems

**Figure 2:**
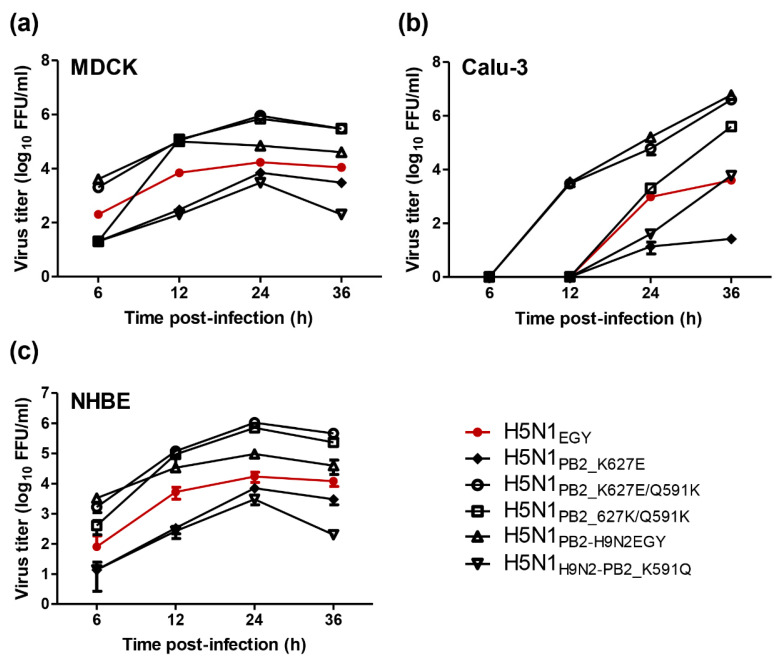
Growth kinetics of wild-type, reassortant, and PB2-mutated viruses in different mammalian systems. MDCK (**a**) and Calu-3 (**b**) were infected with each virus in triplicate with MOI = 0.1. Differentiated NHBE cells (**c**) were infected with the different viruses at MOI = 1. Aliquots from the supernatant were harvested at 6, 12, 24, and 36 h post-infection and titrated.

Mammalian MDCK and Calu-3 were infected with wild-type H5N1_EGY_ and the recombinant variants of interest (Figure 1) at low multiplicity of infection (MOI) = 0.1 and were incubated at 37 °C for 6, 12, 24, and 36 h post-infection. Interestingly, all variant H5N1_EGY_ carrying the 519K alone or in combination with 627K could replicate efficiently when compared to the well-replicating reassortant H5N1_PB2_H9N2EGY_ and the wild-type H5N1_EGY_ strain (Figure 2a,b). In parallel, differentiated NHBE cells were infected with the different viruses at MOI = 1 (Figure 2c). Here, the H5N1_EGY_ variant, carrying the 591K alone or in combination with 627K, replicated efficiently to titers that were comparable to H5N1_PB2-H9N2EGY_ but significantly higher than the wild-type H5N1_EGY_ (Figure 2c). Obviously, all H5N1_EGY_ variants carrying the avian-like adaptive mutations, 591Q and 627E, replicated significantly lower than the wild-type H5N1_EGY_ (Figure 2a–c).

### 3.3. Replication Efficiency of PB2_591K H5N1 Variants Is Associated with Enhanced In Vitro Polymerase Activity

**Figure 3:**
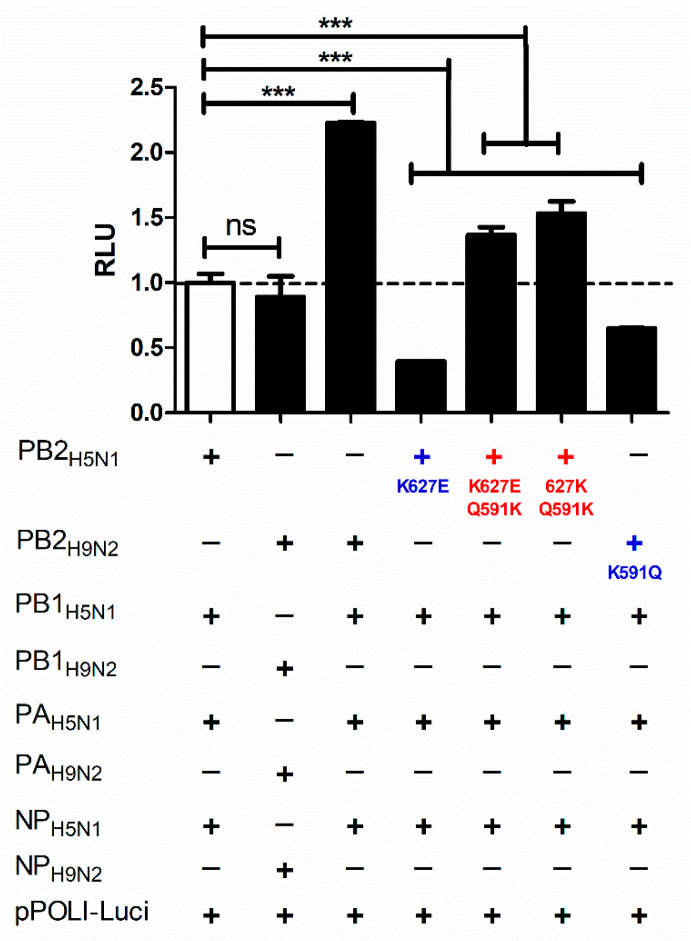
Polymerase activity of parent H5N1, H9N2, and other studied PB2 variants as measured by dual luciferase assay (*n* = 3). The significance was tested using a one-way analysis of variance ANOVA, followed by Dunnett’s multiple comparison post hoc test, and the significant differences are indicated (*** = *p* ≤ 0.001 and non-significant = ns).

To determine whether the increased replication efficiency in mammalian cell culture models of H5N1 variants carrying the PB2-591K, either alone or in combination with 627K, is associated with improved polymerase activity, we compared the vRNP complex activity of the various viruses/variants using a mini-genome reporter assay (Figure 3). Compared to the vRNP activity of H5N1_EGY_, the H5N1_PB2_H9N2EGY_-, H5N1_PB2_K627E/Q591K_-, and H5N1_PB2_627k/Q591K_ vRNP activities were significantly improved. On the other hand, H5N1 variants carrying avian-like mutations showed significantly lower polymerase activities when compared to the wild-type strains, H5N1_EGY_ and H9N2_EGY_ (Figure 3).

## 4. Discussion

Due to the high genetic plasticity based on the error-prone polymerase activity and segmented genome nature, IAV can exhibit functionally adaptive substitutions and can undergo gene segment reassortment among distinct viruses [1,8,9,10]. This enables these viruses to quickly adapt to new host species or selective/antiviral pressures. Therefore, genomic changes can cause dynamic changes in viral characteristics, including their propagation efficiency, polymerase activity, host specificity, and interspecies transmission. This results in the ongoing appearance of unanticipated AIV variants with different chances to be transmitted from birds to humans [1].

**Figure 4:**
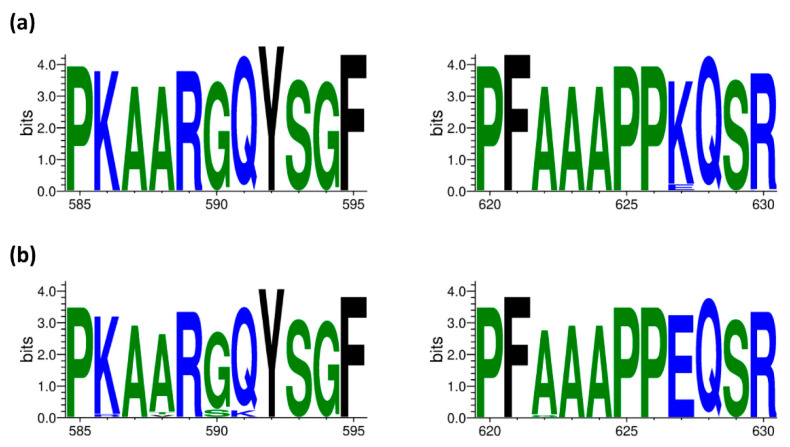
Prevalence of amino acid (aa) residue at positions 591 and 627 among Egyptian H5N1 isolates (2006 to 2017) (**a**) and H9N2 isolates (2011–2021) (**b**). The graphic was created via a Web-based WebLogo application (http://weblogo.threeplusone.com/create.cgi (accessed on 10 November 2022)) [27]. The aa color is given according to their chemical properties.

Genetic shifts and drifts of IAV have historically posed hazards to public health in the form of annual influenza epidemics and occasional influenza pandemic outbreaks [1]. Recently, it was reported that IAV reassortants expressing internal protein-encoding segments from H9N2-subtype AIV could enhance the zoonotic potential of several other AIV subtypes such as H5N1 and H7N9 [1]. In addition, it has previously been revealed that aa substitution in the PB2 (Q591K) is significantly acquired in the circulating H9N2 viruses [24]. By analyzing the available PB2 segments of Egyptian H5N1 isolates from 2006 to 2017 and in H9N2 isolates from 2011 to 2021 in GenBank and GISAID databases, it was remarkable that the prevalence rates of 591K mutations in PB2 proteins of H9N2 isolates and the mutation 627K among Egyptian H5N1 isolates are high (Figure 4).

On the other hand, the genetic marker PB2-627 is well-known to affect host specificity, pathogenicity, and transmission of human H5N1 isolates [25,26]. Along these lines, we recently showed that the genetic reassortment of internal-protein encoding segments from the low pathogenic AIV (LPAIV) H9N2-subtype, specifically the PB2 and PA segments, could enhance the replication efficiency and polymerase activity of the HPAIV H5N1-subtype without altering viral pathogenicity [9]. Interestingly, both recent field isolates H9N2_EGY_ and H5N1_EGY_ encode for PB2 proteins with mammalian-adaptive markers PB2-591K and PB2-627K, respectively [9]. Here, we were interested to elucidate the differential impact of both mutations on the genetic background of HPAIV H5N1-subtype of clade 2.2.1.2 to define the genetic determinant of predefined findings following the introduction of the PB2 segment from H9N2_EGY_ into the H5N1_EGY_ genetic background [9]. Remarkably, H5N1 viruses of this clade are of high concern and were associated with frequent reporting of human cases and mortalities until 2017 [1]. To this point, we constructed a panel of recombinant, reassortant, and mutated viruses with various aa residues at PB2-627 and 591. Intriguingly, these viruses displayed diverse features in terms of replication efficiency and polymerase activities.

Herein, the recombinant H5N1 viruses showed diverse viral replication kinetics in various relevant mammalian and avian cell systems, emphasizing that PB2_627K/Q591K and PB2_K627E/Q591K in the PB2-H5N1 background increase viral replication efficiency in permanent and primary mammalian-type cells, including MDCK, Calu-3, and NHBE. This confirms that the PB2-591K can efficiently enhance the replication of the H5N1_EGY_ virus, either alone or in combination with the PB2-627K. In contrast, the recombinant H5N1_EGY_ viruses that carry PB2_K627E and H9N2-PB2_K591Q show replication performance at a minimal level in mammalian systems. Our results are comparable to Wang and his colleagues who showed similar effects of the Q591K mutation but in the backbone of the H9N2 virus [28].

To determine the impact of naturally-occurring PB2 mutations on viral polymerase activity, they were individually introduced into corresponding PB2 segments at position 627 and 591 and were then tested in transient mini genome systems, showing clearly that the selected mutations, Q591K, K627E/Q591K, and 627K/Q591K, could enhance the viral polymerase activity compared to the corresponding controls. Consistently, Mehle and colleagues showed in a previous work that the pandemic swine-origin human influenza virus H1N1 that appeared in 2009 utilized a similar strategy to transcend the species barrier from pigs to humans [17]. The PB2 positions 590 and 591 have been described to replace the function of PB2-627K. When both the G590S/Q591R and E627K exchanges were present, no synergistic impact was seen, indicating that such mutations constitute a compensating replacement [17].

Taking into consideration the enhanced vRNP polymerase activity in minigenome assays of the H5N1_EGY_ reassortant expressing PB2 from H9N2_EGY_ “H5N1_PB2_H9N2EGY_” compared to the H5N1_PB2_K627E/Q591K_- and H5N1_PB2_627k/Q591K_ highlights the role of other synergistic aa changes present on the PB2 of H9N2_EGY_. Actually, the PB2 of H5N1_EGY_ and H9N2_EGY_ differ by 26 aa, as previously stated [9]. Nevertheless, we could show in this study that the 591K is the main molecular determinant of the enhanced replication efficiency of H5N1_PB2_K627E/Q591K_- and H5N1_PB2_627k/Q591K_ that replicated to higher levels when compared to the parent H5N1_EGY_ and the H5N1_PB2_K627E/Q591K_- and H5N1_PB2_627k/Q591K_ variants.

The host range of influenza viruses is reliant on the compatibility of viral components with the human host [29]. Several investigations have shown that mammalian adaptation in PB2 affects the host factor reliance in both avian and human hosts. For example, it could be demonstrated that the cellular importin-1 and -7 proteins favorably control the polymerase activity and replication of IAV with PB2-E627K in mammalian hosts, whereas importin-3 has a negative effect on all IAV [30].

Despite that, the currently circulating avian H5Nx viruses of clade 2.3.4.4 including H5N8 and H5N1 have shown low or rare zoonotic potential in Egypt and worldwide and limited genetic compatibility with H9N2 viruses; the virulence of these stains was potentiated upon acquisition of the PB2 from G1-like H9N2 viruses [10]. Nevertheless, genetic reassortment of these H5Nx viruses with PB2 segments from G1-like H9N2 Egyptian isolates with 591K might quickly make the situation much worse. To this point, we recommend further investigation of the impact of these mammalian-like markers on the zoonotic potential of H5Nx strains (H5N8 and H5N1) of clade 2.3.4.4.

## 5. Conclusions

Our findings indicate that in the H5N1 virus, the PB2-Q591K mutation either alone or in combination with the PB2-E627K mutation, can strongly affect the viral replication efficiency and polymerase activity in mammalian systems in vitro, potentiating the zoonotic potential of these viruses and probably supporting their pandemic potential. Therefore, close monitoring of the circulating H9N2 and H5N1 with PB2-Q591K is mandatory and must be considered as viruses of concern.

## Figures and Tables

**Table 1:** Site-directed mutagenesis primers to generate mutated PB2 segments.

Segment_Mutation	Primer	Mutagenesis Primer Sequence
H9N2_PB2_K591Q	K591Q_F	5′-CCTAAGGCTGCCAGAGGTCAGTATAGTGGATTTGTGAG-3′
	K591Q_R	5′-CTCACAAATCCACTATACTGACCTCTGGCAGCCTTAGG-3′
H5N1_PB2_K627E	K627E_F	5′-GCAGCAGCCCCACCGGAACAAAGCAGAATG-3′
	K627E_R	5′-CATTCTGCTTTGTTCCGGTGGGGCTGCTGC-3′
H5N1_PB2_K627E	K627E_F	5′-GCAGCAGCCCCACCGGAACAAAGCAGAATG-3′
	K627E_R	5′-CATTCTGCTTTGTTCCGGTGGGGCTGCTGC-3′
H5N1_PB2_591K	591K_F	5′-CCTAAAGCTGCCAGAGGTAAATACAGTGGATTTGTGAG-3′
	591K_R	5′-CCTAAAGCTGCCAGAGGTAAATACAGTGGATTTGTGAG-3′
H5N1_PB2_591K	591K_F	5′-CCTAAAGCTGCCAGAGGTAAATACAGTGGATTTGTGAG-3′
	591K_R	5′-CCTAAAGCTGCCAGAGGTAAATACAGTGGATTTGTGAG-3′

**Table 2:** Genetic composition of parent AIV H5N1, AIV H9N2, and the studied variants of interest (VOC).

RG/Reassortant/Mutant	Genome Composition of Rescued Viruses							
	PB2	PB1	PA	HA	NP	NA	M	NS
H5N1_EGY_	wt (627K)	wt	wt	wt	wt	wt	wt	wt
H9N2_EGY_	wt (591K)	wt	wt	wt	wt	wt	wt	wt
H5N1_PB2-H9N2EGY_	wt (951K)	wt	wt	wt	wt	wt	wt	wt
H5N1_PB2-H9N2EGY_K591Q_	(+) K591Q	wt	wt	wt	wt	wt	wt	wt
H5N1_PB2_K627E_	(+) K627E	wt	wt	wt	wt	wt	wt	wt
H5N1_PB2_K627E/591K_	(+) K627E/591K	wt	wt	wt	wt	wt	wt	wt
H5N1_PB2_627K/591K_	(+) 591K/627K	wt	wt	wt	wt	wt	wt	wt

Blue refers to segments derived from H9N2_EGY_; green refers to H5N1_EGY_; wt refers to wild-type; (+) refers to PB2 segments with specific amino acid (aa) mutation(s).

## Data Availability

Not applicable.
